# Accelerated Aging Method of Performance Attenuation of Crystalline Silicon Photovoltaic Modules Under Full-Spectrum Conditions

**DOI:** 10.3390/ma18071507

**Published:** 2025-03-27

**Authors:** Rui Liu, Xin Li, Ying Liu, Zhuoyuan Zhang, Mingli Wu

**Affiliations:** 1 School of Electrical Engineering, Beijing Jiaotong University, Beijing 100044, China; liuruiliyu@sina.com; 2 China Highway & Transportation Society, Beijing 100080, China; lixin_911@126.com; 3 School of Transportation, Beijing Jiaotong University, Beijing 100044, China; 23120847@bjtu.edu.cn (Y.L.); zyzhang0625@163.com (Z.Z.)

**Keywords:** accelerating aging model, crystalline silicon photovoltaic modules, performance degradation, accumulated radiation exposure

## Abstract

Crystalline silicon photovoltaic modules, when subjected to diverse environmental conditions, undergo progressive performance degradation due to factors such as temperature, humidity, light irradiation, and operational duration. Understanding this degradation is essential for reliably correlating laboratory tests with actual operational performance. This study examines the reduction in power generation capacity resulting from the prolonged interaction of these modules with various environmental factors. We developed an accelerated aging model that simulates real-world conditions in the lab, using multiple doses of environmental factors, full-spectrum conditions, and varying light intensities. The developed model indicates an aging acceleration factor of 143.36, though this factor might be higher in actual conditions. To validate our model, we conducted a series of tests under controlled conditions, specifically at a temperature of 70 °C, humidity level of 60%, and triple the standard incident light intensity. The findings revealed a significant correlation between the accelerated aging model and the long-term performance degradation observed in modules operational for 8–10 years. For polycrystalline silicon modules, the correction coefficient associated with the accelerated aging method was determined to range from 0.3 to 0.5. This study presents a reliable approach for connecting long-term performance projections of photovoltaic modules to laboratory testing, providing critical insights into the operational reliability and degradation patterns of crystalline silicon photovoltaic modules within the industry.

## 1. Introduction

Depending on the durability of the material or device, correlation refers to the ability of artificial aging experiments to converge with actual outdoor experiments or exposure in the use environment [1]. Accelerated aging is a method that uses natural acceleration or artificial experimental methods to significantly shorten the time required for aging processes. Compared to traditional outdoor material or device aging, whether artificial accelerated aging experiments are related to natural aging depends on the changes occurring during exposure [2]. These alterations could manifest as changes in mechanical or apparent properties, such as gloss loss or color shifts; alternatively, they might involve modifications in physical, mechanical, or electrical properties, including alterations in tensile strength and electron migration capability.

At present, in the study of the material aging process, the aging of polymer materials has been studied more [3,4,5]. For example, Shi et al. [6] studied the influence mechanism of environmental factors such as temperature, humidity, oxygen, light, chemical media, microorganisms, etc., on the aging of polymer materials and proposed corresponding anti-aging methods for polymer materials. Krauklis et al. [7] presents modular and multiscale modeling approaches for predicting environmental aging and degradation of polymers and polymer composites. Maraveas et al. [8] found using additives and ultraviolet (UV) absorbers on polymers and polymer composites can extend their lifespan by shielding them from the aging effects of UV radiation. Many scholars conduct indoor and outdoor accelerated aging test research on polymer materials [9,10]. Japan used a WE-SH-2C ultraviolet carbon arc lamp aging test box to test 85 types of paint layers and concluded that 200 h of artificial accelerated aging is equivalent to one year of natural aging in Tokyo [11].

While there has been extensive research on the aging of polymer materials, the study of crystalline silicon photovoltaic module aging is still in its nascent stages. In the photovoltaic industry, the reliability test of photovoltaic modules is currently mainly based on the IEC61215 [12] series of standards and the IEC61730 [13] series of standards, which are the core test content and test methods in the international photovoltaic module performance, safety, and reliability testing process [14,15,16,17]. The environmental reliability tests within the aforementioned test series standards include Highly Accelerated Life Testing (HALT) and Highly Accelerated Stress Screening (HASS). It is worth noting that these tests cannot well reflect the impact of long-term outdoor environment on the performance degradation of photovoltaic modules [16,17,18,19], mainly due to two reasons: (1) the time or number of cycles is too short or too small; (2) only a single environmental factor is considered [20]. In particular, the lack of full-spectrum conditions in these tests limits their ability to accurately simulate the complex light environment that photovoltaic modules experience in real-world outdoor settings. Full-spectrum conditions, which include the complete range of solar radiation from ultraviolet (UV) to infrared (IR), are crucial for understanding the comprehensive effects of light-induced degradation on photovoltaic materials.

Tung et al. [21] pointed out that most crystalline silicon photovoltaic modules can successfully pass the 1000 h constant damp heat (DH) test to measure the durability of the module. However, to ascertain the actual durability of the photovoltaic module through the DH test, the test duration must be at least 3000 h (exceeding 4 months). Li et al. [22] pointed out that if a photovoltaic module is operated in a desert area for 25 years, the total ultraviolet (UV) radiation received on its back is about 275 kWh/m^2^, which is far higher than the UV radiation (60 kWh/m^2^) specified in the IEC 61730 series of standards for testing the power attenuation caused by UV on photovoltaic modules. Nam et al. introduced and analyzed long-term reliability tests for high-power-density photovoltaic (PV) modules by IEC 61215 and light-combined damp heat cycles, such as DIN 75220. The results indicated that post-light soaking procedure, light-combined damp heat cycles caused a 3.51% power drop [23]. There are numerous technological pathways in photovoltaics, with crystalline silicon photovoltaics being the most predominant, accounting for over 98% of the photovoltaic market. However, achieving rapid, accelerated verification of the performance degradation of crystalline silicon photovoltaic modules under laboratory conditions, while comprehensively incorporating all major influencing factors, has consistently posed a challenge within the industry. The integration of full-spectrum conditions into accelerated aging tests is essential for developing more accurate and reliable methods to predict the long-term performance and durability of photovoltaic modules. The key contribution of our study lies in addressing a critical gap in the field of photovoltaic module aging. Our research provides valuable insights into this underexplored area, contributing to the scientific understanding of aging mechanisms specific to crystalline silicon photovoltaic modules.

## 2. Establishment of an Accelerated Performance Aging Model for Crystalline Silicon Photovoltaic Modules with Full-Spectrum Conditions

Under standard laboratory conditions, evaluating the long-term reliability of materials typically requires prolonged experimental periods. However, traditional reliability testing methods often fail to meet the demands of rapid product development and updating cycles, as they are unable to promptly verify high reliability within practical application scenarios. Therefore, it is essential to establish a suitable accelerated testing model that aligns with laboratory conditions, enabling appropriate experiments to be conducted on targeted test objects. Such experiments are defined as “accelerated testing” [24].

Photovoltaic modules are long-term application products typically designed for 25 years or even longer. However, the replacement of new products and technologies of photovoltaic modules is also very fast. Completing extensive outdoor life verification or conventional laboratory testing procedures within the short market introduction cycle of new modules is challenging. Therefore, establishing accelerated testing methods for crystalline silicon photovoltaic modules under laboratory conditions becomes essential. This approach draws upon extensive accumulated experience regarding material performance degradation to effectively simulate and predict module performance degradation, enabling quicker and more efficient reliability assessments.

In addition to being affected by long-term temperature and humidity changes in the natural working environment, crystalline silicon photovoltaic modules are also affected by the electric field microenvironment inside the cell [25]. Therefore, these primary influencing factors need to be considered when establishing the corresponding module accelerated aging model.

The Arrhenius model is the most typical and widely used acceleration model for describing the accelerated aging process of material properties [24]. It incorporates thermal and voltage acceleration (as shown in Formula (1)), which can be applied either independently or in combination. This model is extensively employed to establish the relationship between product lifetime and temperature, effectively capturing how temperature affects product reliability under specific application conditions. The mathematical expression of the Arrhenius model is as follows:

(1) $$AF=\exp[\frac{\mathrm{Ea}}{K}\times (\frac{1}{T_{u}}-\frac{1}{T_{t}})]$$

The Arrhenius model only considers the thermal acceleration factor, so in the model, only $T_{t}$ and $T_{u}$ parameters are used as parameters of temperature influencing factors. Based on this model, the Arrhenius model [16] (Arrhenius Mode with Humidity) after further considering the influence of humidity factors is expressed as shown in Formula (2). This model holistically accounts for the impacts of temperature and humidity. In comparison to the Arrhenius model, it offers a more precise depiction of the accelerated test model under combined temperature and humidity conditions. Its expression is as follows:

(2) $$AF={\left(\frac{RH_{t}}{RH_{u}}\right)}^{3}\cdot \exp[\frac{\mathrm{Ea}}{K}\times (\frac{1}{T_{u}}-\frac{1}{T_{t}})]$$

The physical meanings of each parameter in the above Formula (2) are as follows:

AF—the acceleration factor;

Ea—the energy consumed by the precipitation failure, also known as the activation energy of the failure reaction, with a value between 0.3 and 1.2 eV;

K—Boltzmann constant, with a value of 8.617385 × 10^−5^ eV/K;

$T_{u}$—the absolute temperature value under the use conditions (under non-accelerated state), with K (Kelvin) as the unit;

$T_{t}$—the absolute temperature value under the test conditions (under accelerated state), with K (Kelvin) as the unit;

$RH_{u}$—the relative humidity value under the use conditions (under non-accelerated state);

$RH_{t}$—the relative humidity value under the test conditions (under accelerated state).

Within this context, the activation energy *Ea* represents the energy required to induce atoms within the material’s crystal lattice to transition from one equilibrium position to another relative equilibrium position. Ea is commonly different [16,24,26,27,28]. Table 1 lists the range of activation energies for different materials:

There is currently no recognized accelerated aging test model for crystalline silicon photovoltaic modules that incorporates light radiation conditions into the acceleration factor [29]. However, to quantitatively compare the relationship between laboratory accelerated aging tests and outdoor natural exposure to light radiation, an environmental factor affecting light radiation, the literature [16,26,27] proposed a module performance aging model caused by light radiation, which has certain reference significance. The expression is shown as Formula (3):

(3) $$AF=\gamma \cdot \frac{s_{0}\cdot \left(a+b\frac{s}{s_{1}}\right)\cdot t}{A_{1}\cdot A\cdot T}$$

In this formula, the physics meaning of each parameter is listed as follows:

AF—the acceleration factor;

γ—correction coefficient of the laboratory acceleration rate model;

$S_{0}$—astronomical radiation intensity;

S—actual hours of solar exposure;

$S_{1}$—permitted hours of exposure;

t—actual exposure time;

$A_{1}$—laboratory simulated irradiation intensity coefficient;

A—laboratory simulated irradiation intensity;

T—light radiation acceleration test time.

Based on the above description, photovoltaic modules experience performance degradation primarily due to the combined effects of temperature, humidity, and irradiation in real-world conditions. The accelerated aging model after introducing these three factors can be summarized as shown in Formula (4):

(4) $$AF^{\prime }=AF\cdot AF_{RH}\cdot AF_{SR}=\exp[\frac{\mathrm{Ea}}{K}\times (\frac{1}{T_{u}}-\frac{1}{T_{t}})]\cdot {\left(\frac{RH_{t}}{RH_{u}}\right)}^{3}\cdot \gamma \cdot \frac{s_{0}\cdot (a+b\frac{s}{s_{1}})t}{A_{1}\cdot AT}$$

## 3. Study on the Strict Test Conditions of Factors Affecting the Accelerated Aging of Crystalline Silicon Photovoltaic Modules

To determine the test dose during the accelerated aging laboratory test (i.e., the acceleration factor of each influencing factor), we intensified the test of the conventional component performance test standard sequence (IEC 61215 [12]). The current practical applications in the photovoltaic industry generally fall within this range, thus reflecting real-world conditions.

### 3.1. Multiple Thermal Cycle (TC) Tests 

#### 3.1.1. Sample Preparation

Seven monocrystalline silicon PERC modules with a module power of 415 W were selected.

#### 3.1.2. Test Content

Test sequence 1: Four groups of crystalline silicon photovoltaic modules completed TC50 + TC50 + TC50. After every 50 thermal cycle tests, the module power test was performed; the displayed value is the module power attenuation rate.

Test sequence 2: Another three groups of crystalline silicon photovoltaic modules were subjected to TC cycle tests with increased doses. The test conditions were TC200 + TC200 + TC200. After every 200 thermal cycle tests, the module power test was performed. The module power was measured and compared with the initial module power. The displayed value was the module power attenuation rate.

#### 3.1.3. Experimental Results and Discussion

**Figure 1:**
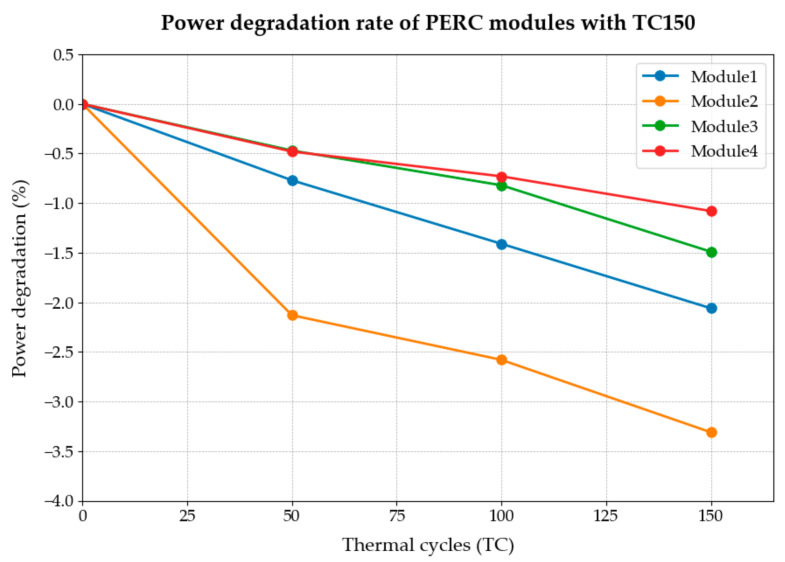
Power degradation rate of PERC modules with TC150.

The performance test results (performance degradation rate) of sequence 1 are shown in Table 2 and Figure 1.

Generally, the standard IEC 61215 [12] specifies TC150 as the number of thermal cycles for testing, corresponding exactly to test sequence 1 in this study. With continuous advancements in module technology and manufacturing processes, traditional test requirements have lagged the needs of industrial development to a certain extent. To identify a suitable thermal cycle failure point for contemporary mainstream PERC components and simultaneously establish a reasonable comprehensive test process thermal cycle test point, this study has developed two test protocols to comparatively examine the characteristics of component performance degradation resulting from temperature cycling.

This study quantified module performance degradation after each thermal cycling interval using the power attenuation rate. Initially, performance data for PV modules were recorded before exposure to thermal cycling. Subsequently, module performance was measured after completing TC50, TC100, and TC150 cycles, allowing for comparative analysis at each stage.

Figure 1 illustrates the power attenuation results for crystalline silicon photovoltaic modules subjected to the TC150 test. The data clearly indicate a continuous decline in module power with an increasing number of thermal cycles. However, examining the power trends of Modules 1, 3, and 4 reveals that traditional TC150 testing conditions inadequately characterize the thermal cycling degradation behavior of crystalline silicon PERC modules. Specifically, the power degradation exhibited by these modules indicates that the IEC standard may not fully capture the stabilization or saturation behavior within the 150-cycle limit. Thus, a new testing approach, denoted as test sequence 2, was proposed and implemented in this study.

**Figure 2:**
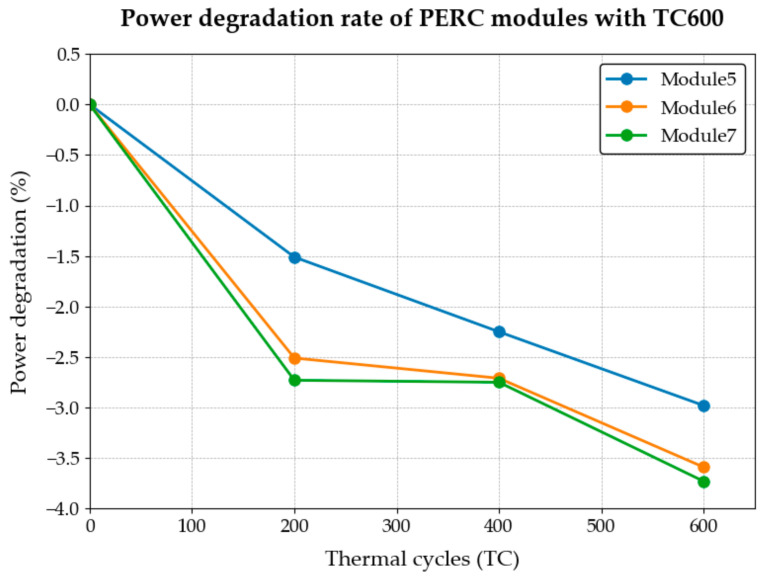
Power degradation rate of PERC modules with TC600.

The performance test results (performance degradation rate) of sequence 2 are shown in Table 3 and Figure 2.

Figure 2 illustrates the fluctuations in power degradation experienced by crystalline silicon photovoltaic modules subsequent to the TC600 test. As illustrated in the figure, the three groups of photovoltaic modules (Module 5, Module 6, and Module 7) indicate that the modules have undergone significant performance degradation following 200 thermal cycles (TC200), with the rate of degradation now beginning to decelerate. Therefore, under the condition of a single thermal cycle action parameter, TC200-TC400 can be adopted as the appropriate dose range for future comprehensive accelerated testing scenarios.

### 3.2. Multiple Humidity–Freeze (HF) Aging Tests 

#### 3.2.1. Sample Preparation

Four monocrystalline silicon PERC modules with a module power of 415 W were selected.

#### 3.2.2. Test Content

The test sequence was HF10 + HF10 + HF10. After every 10 wet–freeze tests, perform a module power test.

#### 3.2.3. Experimental Results and Discussion

**Figure 3:**
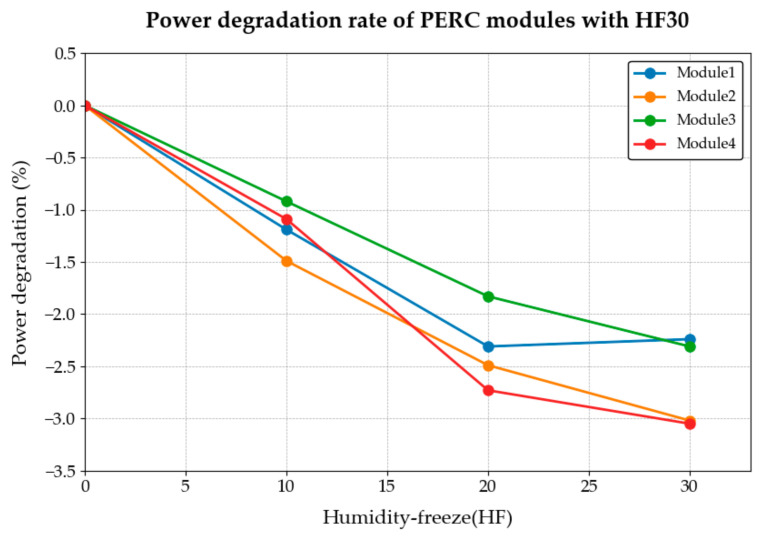
Power degradation rate of PERC modules with HF30.

The performance test results (performance degradation rate) are shown in Table 4 and Figure 3.

The environmental characteristics of high- and low-temperature environments and humidity will greatly accelerate the aging rates of components and materials. It can also be seen from Figure 3 that with the increase in the number of HF test cycles, the PV modules’ power degradation rate changes. Although the IEC 61215 standard specifies the HF10 test, Figure 3 indicates that this number of cycles is insufficient for effectively identifying a stable inflection point in module performance degradation. When the HF test was carried out 20 times (i.e., HF20), the test results showed that the power reduction rate of the four modules was still stable at about 1.5%, and its power decreasing maintained a relatively stable linear reduction process. When the number of HF experiments continued to increase to 30 times, we found that the performance reduction rate of photovoltaic modules was reduced to about 0.5%. Specifically, at approximately HF20, the degradation rate of crystalline silicon photovoltaic modules significantly slowed, indicating a clear stabilization point. Therefore, this study uses HF20-HF30 times as a stricter test condition to distinguish the quality of modules. This dose is about 2–3 times the current standard dose.

Based on the above analysis of the IEC 61215 multiple tests, the existing standards need to be strengthened and improved according to the aging trend and rate analysis. Therefore, UV radiation at 60 kWh/m^2^, 200 thermal cycles (TC200), and 20–30 humidity–freeze cycles (HF20-30) tests can be used as tightened test standard conditions to distinguish the quality of the PV modules. In summary, the selection of multiple doses is controlled at 2~4 times the dose under the IEC 61215 test conditions. At the same time, this project ultimately defines the test boundary of light intensity to 3000 W/m^2^ (that is, three times the irradiation intensity (full-spectrum) of the crystalline silicon laboratory test conditions).

According to the formulas of temperature factor, humidity factor, and irradiance factor in the comprehensive accelerated aging model of photovoltaic modules [16,24,26,27], it is evident that during laboratory testing, as the test temperature, humidity, and irradiance increase, the comprehensive acceleration factor also increases, reflecting the increase in the accelerated aging rate, and the long-term outdoor degradation of photovoltaic modules can be predicted in a shorter time. Considering the realistic constraints posed by outdoor extreme conditions, this study proposes elevated laboratory conditions: temperatures, humidity, and irradiance levels are intensified appropriately. The resulting comprehensive acceleration factors for module aging can be calculated, allowing reliable prediction of long-term outdoor degradation behavior.

The acceleration factors are calculated using the following expressions:

Temperature factor: AF = 6.425;

Humidity factor: AFRH = 4.291;

Irradiance factor: AFSR = 5.20 (where γ is temporarily set to 1);

Comprehensive accelerated aging factor: AF′ = 143.35.

It is calculated that under the accelerated aging conditions of irradiance 3000 W/m^2^, temperature 70 °C, and relative humidity 60%, the comprehensive accelerated aging ratio of the component calculated based on the model is 143.35.

## 4. Test Results and Discussion

To verify the impact of temperature, humidity, and accumulated irradiation on photovoltaic module performance under triple the standard incident light intensity, the following experiment was designed.

### 4.1. Experimental Conditions

Irradiation intensity: 3000 W/m^2^;

Relative humidity: 65%;

Test temperature: 70 °C;

Cumulative irradiation: 0, 100, 200, 300, 400, 500, 600, 700, 800 (kWh/m^2^).

### 4.2. Sample Preparation

To validate the developed accelerated aging model and its experimental results, a total of 11 samples were prepared, which is as follows: The test PV module samples involved various cell arrangement samples, such as 6 × 10, 6 × 6, and 2 × 3, with the total number of cells ranging from 6 to 60 pcs. The initial performance and components are shown in Table 5. At the same time, considering the comprehensiveness of the degradation research of various components, various types of photovoltaic cells were selected, including conventional single and multi-crystalline, PERC single crystal, black silicon multi-crystalline, N-type bifacial, and other types of components, as well as new photovoltaic components such as single crystal PERC [30]. To ensure broad applicability and representativeness across the photovoltaic manufacturing industry, samples were sourced from at least two different manufacturers.

### 4.3. Experimental Results and Discussion

Given the diversity of photovoltaic module types and sizes in the experiment, the attenuation percentage method was used for the measurement data results to determine and analyze the attenuation degree of various modules during the accelerated attenuation process. The experimental results are shown in Table 6.

**Figure 4:**
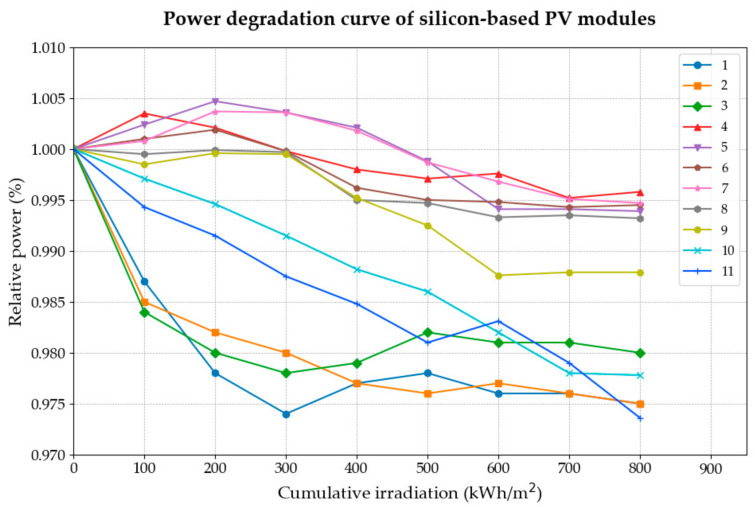
Power degradation curve of silicon-based PV modules under cumulative irradiation conditions.

Under the irradiation intensity of 3000 W/m^2^, the response power of PV modules under STC conditions (1000 W/m^2^, 25 °C in temperature, AM1.5) was measured at cumulative irradiation of 100, 200, 300, 400, 500, 600, 700, and 800 kWh/m^2^, and the results after taking the average of each group are shown in Table 5. To more comprehensively investigate the performance variations of the components under different irradiation accumulation conditions, based on the data provided in the table above, this study constructed the performance change diagram for the aforementioned 11 groups of photovoltaic modules under varying cumulative irradiation conditions, as illustrated in Figure 4.

As shown in Figure 4, while certain samples exhibited a slight initial increase in power, most samples experienced significant overall degradation as cumulative irradiation increased. At the same time, as the exposure time increases—that is, the cumulative radiation amount increases—the power of almost all samples shows a significant decrease. Specifically, in the experiment, when the cumulative irradiation was 0~300 kWh/m^2^, the power of most samples showed an obvious decline, which is a rapid reduction process. Among the samples, the power reduction of samples 1, 2, and 3 was the most rapid, as they belong to the polycrystalline PV modules category. This was followed by samples 5, 10, and 11, which also exhibited relatively quick attenuation. The power reduction rate for these samples during this stage ranged from 0.5% to 2.75%.

All samples exhibited a noticeable power reduction once the cumulative irradiation reached 400 kWh/m^2^; however, except for samples 10 and 11, the reduction rate demonstrated a stable attenuation pattern. As the irradiation accumulated from 400 kWh/m^2^ to 800 kWh/m^2^, although all the aforementioned samples continued to experience power reduction, each sample displayed a relatively moderate attenuation process. As illustrated in the figure, apart from sample No. 7 (approximately 0.6%), sample No. 10 (approximately 1%), and sample No. 11 (approximately 0.91%), which exhibited attenuation rates exceeding 0.6%, the attenuation of all other samples generally fell within the range of 0.1% to 0.2%. This process demonstrates that the power reduction of crystalline silicon photovoltaic modules is directly correlated with the increase in cumulative radiation under identical environmental conditions.

## 5. The Power Reduction of Crystalline Silicon Photovoltaic Modules Under Outdoor Application Conditions

To compare the results of the laboratory’s accelerated aging test method for crystalline silicon photovoltaic modules, the research team also investigated and extracted 63 sets of polycrystalline modules that had been in operation for 8–10 years in some power stations of some owners in China. Given the technological constraints of that era, the empirical modules collected on-site were PV modules. After many years of operation, the power change results at the time of sampling are shown in Table 7.

**Figure 5:**
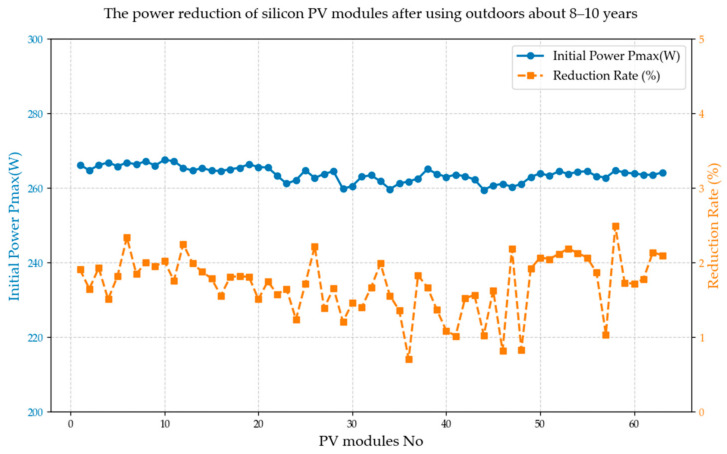
The power reduction of silicon PV modules after using outdoors about 8–10 years.

As indicated in Table 6, the power reduction range of 63 photovoltaic modules following approximately 8–10 years of field application spans from about 0.71% to 2.49%. Among these, module #36 exhibits the smallest power attenuation, while module #58 shows the largest. The average reduction rate for all sampled crystalline silicon photovoltaic modules is 1.71%. On an annual basis, the average annual reduction rate is estimated to be between approximately 0.171% and 0.214%. The overall reduction trend is depicted in Figure 5.

By comparing the performance degradation observed in outdoor environments to that obtained under accelerated laboratory irradiation, it was determined that a cumulative irradiation of approximately 300 kWh/m^2^ in the laboratory corresponds to the degradation typically experienced by polycrystalline photovoltaic modules after long-term outdoor exposure for 8 years. Based on this correlation, a relationship between cumulative irradiation in real-world conditions and corresponding laboratory irradiation was established, allowing calculation and adjustment of the acceleration factor. Consequently, under laboratory accelerated conditions, the comprehensive acceleration factor for polycrystalline photovoltaic modules was calculated as 143.35 (AF = 143.35). Based on the laboratory conditions, when the exposure time of photovoltaic modules is 100 h, the cumulative radiation of the module is as follows:

(5) $$Q=P\times T=3000W/m^{2}\times 100h=300\mathrm{kW}\cdot h/m^{2}$$

Combined with the accelerated aging factors, the total cumulative radiation can be calculated as follows:

(6) $$M=Q\times AF’=300\mathrm{kW}\cdot h/m^{2}\times 143.35=43005\mathrm{kW}\cdot h/m^{2}$$

If such an amount of radiation is in the natural environment, we can estimate the PV module applied time as follows:

(7) $$D=M/24=43005/\left(6\mathrm{or}10\right)=\left(7167.5\mathrm{or}4300\right)\mathrm{day}=\left(19.64\mathrm{or}11.67\right)\mathrm{year}$$

where the number 24 means 24 h a day, and the effective irradiation time in a day is about 6 to 10 h. One possible explanation is that while the PV module’s equivalent rated power time is approximately 6 h per day, its actual exposure to sunlight can extend up to 10 h. Consequently, we derived the aforementioned range.

Table 8 shows the corresponding time for equivalent natural application scenarios calculated based on the results of the laboratory full-spectrum three times light intensity cumulative irradiance calculation.

Since there are relatively few single-crystal photovoltaic power stations with over 10 years of field-available data, there is currently no corresponding correction work on the accelerated aging factor of single-crystal modules. Further research will continue to investigate the long-term performance of single-crystalline photovoltaic modules.

## 6. Conclusions

This paper identifies a significant performance inflection point in crystalline silicon photovoltaic modules by increasing the test dose by 2–3 times under multiple IEC 61215 test conditions. Based on this finding, a research plan was developed to investigate the impact of cumulative irradiation on module performance under full-spectrum light with three times the standard irradiance while maintaining controlled temperature and humidity conditions. Experimental results demonstrate that tripling the incident light intensity does not cause thermal damage to the photovoltaic modules. Instead, it effectively accelerates cumulative irradiation, enabling a faster evaluation of long-term performance degradation.

The laboratory method of applying cumulative irradiation under multiple incident light intensities across the full spectrum proves to be an effective approach for assessing the degradation process of photovoltaic modules. By integrating temperature, humidity, and electric field conditions, an accelerated aging model was established. The calculated accelerated aging factor for photovoltaic modules under three times light intensity in laboratory conditions was 143.35. When compared with real-world performance data, a correction coefficient of 0.3–0.5 was applied, resulting in an adjusted acceleration factor of 43–71.68 for polycrystalline silicon modules.

From this, the correlation between cumulative irradiation under laboratory conditions and real-world operational hours was determined. This approach provides a robust method for verifying the long-term performance degradation of photovoltaic modules, offering a reliable basis for rapidly assessing performance attenuation throughout the module’s entire lifecycle.

In future research, we aim to enhance our understanding of module degradation by gradually incorporating additional influencing factors and expanding our testing scope to include high salinity, dirt accumulation, and mechanical impacts, in accordance with relevant standards. We will conduct experiments to gain new insights into module performance under diverse environmental stresses. Furthermore, beyond crystalline silicon photovoltaic modules, we plan to test mainstream photovoltaic technologies such as Copper Indium Gallium Selenide (CIGS) thin-film and perovskite modules, utilizing appropriate methods and models to address their accelerated aging challenges.

## Figures and Tables

**Table 1:** The range of activation energies for different materials.

Materials	Activation Energy (Ea)
Oxide film	0.3 eV
Ionicity (Na ion drift in SiO_2_)	1.0~1.4 eV
Ionicity (slow collapse of Si-SiO_2_ interface)	1.0 eV
Electron migration disconnection	0.6 eV
Aluminum corrosion	0.6~0.9 eV
Intermetallic compound growth	0.5~0.7 eV

**Table 2:** Power degradation rate of PERC modules with TC150.

No.	Power Degradation (%)		
	TC50	TC100	TC150
Module 1	0.77	1.41	2.06
Module 2	2.13	2.58	3.31
Module 3	0.47	0.82	1.49
Module 4	0.48	0.73	1.08

**Table 3:** Power degradation rate of PERC modules with TC600.

No.	Power Degradation (%)		
	TC200	TC400	TC600
Module 5	1.51	2.25	2.98
Module 6	2.51	2.71	3.59
Module 7	2.73	2.75	3.73

**Table 4:** Power degradation rate of PERC modules with HF30.

No.	Modules Power Degradation Rate (%)		
	HF10	HF20	HF30
Module 1	1.19	2.31	2.24
Module 2	1.49	2.49	3.02
Module 3	0.92	1.83	2.31
Module 4	1.09	2.73	3.05

**Table 5:** Samples and their initial power for accelerated aging testing.

No.	Specification	Battery Size	Type	Quantity (Pcs)	Power (W)
1	6 cell × 10 cell	156 mm × 156 mm	Polycrystalline	6	260
2	6 cell × 6 cell	156 mm × 156 mm	Polycrystalline	6	150
3	3 cell × 3 cell	156 mm × 156 mm	Polycrystalline	20	38
4	3 cell × 3 cell	156 mm × 156 mm	Monocrystalline	20	40
5	3 cell × 3 cell	156 mm × 156 mm	PERC Monocrystalline	20	50
6	3 cell × 3 cell	156 mm × 156 mm	N-type Monocrystalline	20	50
7	3 cell × 3 cell	156 mm × 156 mm	Monocrystalline	20	45
8	3 cell × 3 cell	156 mm × 156 mm	Monocrystalline	20	40
9	3 cell × 3 cell	156 mm × 156 mm	Monocrystalline	20	40
10	3 cell × 3 cell	156 mm × 156 mm	Monocrystalline	20	40
11	3 cell × 3 cell	156 mm × 156 mm	Monocrystalline	20	40
Total				192	

**Table 6:** Power degradation rates of 11 groups of samples after different cumulative irradiation under the same temperature and humidity conditions.

No.	Cumulative Radiation Exposure (kWh/m^2^)									
	0		100	200	300	400	500	600	700	800
1	Polycrystalline	l	0.987	0.978	0.974	0.977	0.978	0.976	0.976	0.975
2		1	0.985	0.982	0.98	0.977	0.976	0.977	0.976	0.975
3		1	0.984	0.98	0.978	0.979	0.982	0.981	0.981	0.980
4	Monocrystalline	1	1.0035	1.0021	0.9998	0.9980	0.9971	0.9976	0.9952	0.9958
5		1	1.0024	1.0047	1.0036	1.0021	0.9988	0.9941	0.9941	0.9939
6		1	1.0010	1.0019	0.9998	0.9962	0.9950	0.9948	0.9943	0.9945
7		1	1.0008	1.0037	1.0036	1.0018	0.9987	0.9968	0.9951	0.9947
8		1	0.9995	0.9999	0.9997	0.9950	0.9947	0.9933	0.9935	0.9932
9		1	0.9985	0.9996	0.9995	0.9952	0.9925	0.9876	0.9879	0.9879
10		1	0.9971	0.9946	0.9915	0.9882	0.9860	0.9820	0.9780	0.9778
11		1	0.9943	0.9915	0.9875	0.9848	0.9810	0.9831	0.9790	0.9736

**Table 7:** The performance of silicon PV modules after 8–10 years using.

No.	Initial Power Pmax (W)	8 Years Using Pmax (W)	Reduction Value (W)	Reduction Rate
1	266.19	261.11	5.08	1.91%
2	264.82	260.45	4.37	1.65%
3	266.16	261.02	5.14	1.93%
4	266.89	262.86	4.03	1.51%
5	265.89	261.04	4.85	1.82%
6	266.84	260.59	6.25	2.34%
7	266.39	261.47	4.92	1.85%
8	267.2	261.86	5.34	2.00%
9	266.07	260.88	5.19	1.95%
10	267.58	262.18	5.4	2.02%
11	267.25	262.54	4.71	1.76%
12	265.43	259.47	5.96	2.25%
13	264.7	259.44	5.26	1.99%
14	265.42	260.44	4.98	1.88%
15	264.69	259.96	4.73	1.79%
16	264.6	260.47	4.13	1.56%
17	265.05	260.25	4.8	1.81%
18	265.49	260.67	4.82	1.82%
19	266.4	261.57	4.83	1.81%
20	265.65	261.64	4.01	1.51%
21	265.55	260.89	4.66	1.75%
22	263.32	259.17	4.15	1.58%
23	261.21	256.91	4.3	1.65%
24	262.13	258.87	3.26	1.24%
25	264.81	260.26	4.55	1.72%
26	262.71	256.89	5.82	2.22%
27	263.85	260.17	3.68	1.39%
28	264.5	260.11	4.39	1.66%
29	259.81	256.66	3.15	1.21%
30	260.58	256.77	3.81	1.46%
31	263.05	259.37	3.68	1.40%
32	263.46	259.07	4.39	1.67%
33	261.83	256.63	5.2	1.99%
34	259.75	255.71	4.04	1.56%
35	261.24	257.69	3.55	1.36%
36	261.81	259.94	1.87	0.71%
37	262.57	257.76	4.81	1.83%
38	265.1	260.68	4.42	1.67%
39	263.84	260.23	3.61	1.37%
40	263	260.14	2.86	1.09%
41	263.61	260.93	2.68	1.02%
42	263.19	259.2	3.99	1.52%
43	262.34	258.21	4.13	1.57%
44	259.55	256.88	2.67	1.03%
45	260.74	256.5	4.24	1.63%
46	261.12	258.97	2.15	0.82%
47	260.37	254.66	5.71	2.19%
48	261.1	258.92	2.18	0.83%
49	263.01	257.96	5.05	1.92%
50	263.95	258.49	5.46	2.07%
51	263.34	257.95	5.39	2.05%
52	264.54	258.94	5.6	2.12%
53	263.81	258.04	5.77	2.19%
54	264.37	258.75	5.62	2.13%
55	264.53	259.06	5.47	2.07%
56	263.2	258.29	4.91	1.87%
57	262.83	260.1	2.73	1.04%
58	264.72	258.13	6.59	2.49%
59	264.23	259.66	4.57	1.73%
60	263.88	259.35	4.53	1.72%
61	263.65	258.96	4.69	1.78%
62	263.51	257.86	5.65	2.14%
63	264.22	258.68	5.54	2.10%
average value	263.851	259.338	4.51	1.71%

**Table 8:** The relationship between cumulative laboratory irradiation and corresponding cumulative years after introducing acceleration factors (polycrystalline silicon modules).

Item		Cumulative Radiation Exposure (kW·h/m^2^)							
lab	Cumulative radiation exposure	100	200	300	400	500	600	700	800
	Temperature	70 °C	70 °C	70 °C	70 °C	70 °C	70 °C	70 °C	70 °C
	Humidity	60%	60%	60%	60%	q60%	60%	60%	60%
Accelerating factor		143.35							
Correction factor		0.3–0.5							
Correction accelerating factor		43–71.68							
Corresponding cumulative time (year)		3.27	6.54	9.82	13.08	16.35	19.62	22.89	26.16

## Data Availability

The original contributions presented in this study are included in the article. Further inquiries can be directed to the corresponding author.
